# Nutritional and developmental status among 6- to 8-month-old children in southwestern Uganda: a cross-sectional study

**DOI:** 10.3402/fnr.v60.30270

**Published:** 2016-05-27

**Authors:** Grace K. M. Muhoozi, Prudence Atukunda, Robert Mwadime, Per Ole Iversen, Ane C. Westerberg

**Affiliations:** 1 Department of Nutrition, Institute of Basic Medical Sciences, University of Oslo, Oslo, Norway; 2 Department of Human Nutrition and Home Economics, Kyambogo University, Kampala, Uganda; 3 School of Food Technology, Nutrition and Bioengineering, Makerere University, Kampala, Uganda; 4 Department of Hematology, Oslo University Hospital, Oslo, Norway; 5 Institute of Health Sciences, Kristiania University College, Oslo, Norway

**Keywords:** child development, child growth, Uganda, undernutrition

## Abstract

**Background:**

Undernutrition continues to pose challenges to Uganda's children, but there is limited knowledge on its association with physical and intellectual development.

**Objective:**

In this cross-sectional study, we assessed the nutritional status and milestone development of 6- to 8-month-old children and associated factors in two districts of southwestern Uganda.

**Design:**

Five hundred and twelve households with mother–infant (6–8 months) pairs were randomly sampled. Data about background variables (e.g. household characteristics, poverty likelihood, and child dietary diversity scores (CDDS)) were collected using questionnaires. Bayley Scales of Infant and Toddler Development (BSID III) and Ages and Stages questionnaires (ASQ) were used to collect data on child development. Anthropometric measures were used to determine *z*-scores for weight-for-age (WAZ), length-for-age (LAZ), weight-for-length (WLZ), head circumference (HCZ), and mid-upper arm circumference. Chi-square tests, correlation coefficients, and linear regression analyses were used to relate background variables, nutritional status indicators, and infant development.

**Results:**

The prevalence of underweight, stunting, and wasting was 12.1, 24.6, and 4.7%, respectively. Household head education, gender, sanitation, household size, maternal age and education, birth order, poverty likelihood, and CDDS were associated (*p*<0.05) with WAZ, LAZ, and WLZ. Regression analysis showed that gender, sanitation, CDDS, and likelihood to be below the poverty line were predictors (*p*<0.05) of undernutrition. BSID III indicated development delay of 1.3% in cognitive and language, and 1.6% in motor development. The ASQ indicated delayed development of 24, 9.1, 25.2, 12.2, and 15.1% in communication, fine motor, gross motor, problem solving, and personal social ability, respectively. All nutritional status indicators except HCZ were positively and significantly associated with development domains. WAZ was the main predictor for all development domains.

**Conclusion:**

Undernutrition among infants living in impoverished rural Uganda was associated with household sanitation, poverty, and low dietary diversity. Development domains were positively and significantly associated with nutritional status. Nutritional interventions might add value to improvement of child growth and development.

Undernutrition continues to pose challenges to Uganda's children (1). Inadequate dietary intake of nutritious food and infections including diarrheal diseases are major causes of growth faltering among infants (2–4). Undernutrition may start as early as during foetal life as a consequence of inadequate food intake of the mother as well as strenuous work during pregnancy (5–9). This causes growth faltering among the infants, which is often accelerated when complementary feeding starts and low-nutrient-density foods are used to replace breast milk (10). Inadequate food intake also results from low meal frequency. In western Uganda, 34% of children aged 6–23 months are fed only twice or less in a day (11) instead of the recommended three to four times a day (12).

Stunting in Uganda has been linked with low socio-economic status and associated factors such as poor health, sanitation and lack of appropriate knowledge on health and nutrition, gender disparities, low or no education of mothers, and suboptimal infant feeding practices (13, 14). Poor sanitary conditions often lead to infections that increase children's risk of entering a vicious cycle of infection, undernutrition, and impaired linear growth as a result of lack of appetite, increased nutrient needs, and malabsorption (11).

Stunting in early life (0–2 years) has been linked to impaired cognitive, language, and motor development (15–17). However, improved nutrition and catch-up growth have been shown to improve or normalize development after stunting (15, 16, 18). Significant growth and development of the brain occur in the last trimester of pregnancy until two years of age (19, 20). Adequate macro- and micronutrient supply is essential for the growth and maintenance of brain tissue to support cognitive and social development (21, 22). Thus, nutrition indirectly affects children's behaviour and experiences (23). Impaired cognitive function consequently affects the individual's ability to live a productive life and educational attainment (24).

In Uganda, few studies have addressed nutrition, growth, and cognitive development. In the Ugandan Nutrition and Early Child Development Project, communities received media messages on child stimulation, positive parenting, health, hygiene, and nutrition (25). The results indicated improvement in breastfeeding practices, growth rates in the youngest children, and parental attitudes and behaviour supporting early child development, but little effect on the cognitive development of children aged 3.5–6 years (25). An intervention study in rural northern Uganda addressing maternal psychological well-being, child growth, and development, with a nutrition component (dietary diversity and demonstrations of food quantities), showed improvement in cognitive and language scores using the Bayley Scales of Infant and Toddler Development (BSID III). However, there was no improvement in length-for-age *z*-scores (LAZ) (26).

The southwestern part of Uganda, including the Kabale and Kisoro districts, has been known to be nutritionally insecure (11), and the levels of stunting, underweight, and wasting were 41.7, 15.5, and 4.9% respectively, in 2011 (1). In this study, two questions were thus addressed: 1) what factors are associated with nutritional status among 6- to 8-month-old infants in Kabale and Kisoro; and 2) what is the relation between their nutritional status, cognitive, language, and motor development? This study focused on 6- to 8- month-old infants because complementary feeding is recommended to start at 6 months (12, 27), a critical time period when adequate nutrition is key in supporting cognitive and social development (21, 22).

## Subjects and methods

### Study area

The study was conducted in Kabale and Kisoro because of the high levels of stunting. Town centres were excluded to minimize differences in socio-economic status and feeding practices. Inhabitants were predominantly small-scale farmers cultivating small plots of land. Both districts were densely populated with populations of 534,160 (28) and 287,179 (29), respectively.

### Study design and participants

The cross-sectional study was conducted between October 2013 and February 2014. The sampling unit was a household with a lead caregiver as the respondent and an infant aged 6–8 months. Households were excluded if the child had 1) congenital malformation(s), 2) a physical handicap that would influence growth or preclude anthropometric measurements, or influence nutrient intake, or 3) been diagnosed with mental or brain illness as evidenced by the child's mother or a health worker.

### Sample size determination and sampling strategy

Sample size calculations were based on a prevalence of 35% for stunting among 6- to 8-month-old children in southwestern Uganda (1). Power was set at 0.8 with a significance level of 5%. To allow for a precision of±5% and a 30% dropout rate, 500 households were required for the study.

Basing on the 2002 housing and population census, an average of six households per 150 in every village was assumed to have an infant between 6 and 8 months old. Furthermore, the included households were stratified according to the districts. Proportionate simple random sampling was used to select sub-counties to participate in the study (six sub-counties in Kabale and four in Kisoro). Using the list of all villages, nine villages were randomly selected in each of the participating sub-counties. Finally, all consenting households with children aged 6–8 months within a participating village were recruited. If a household had more than one eligible child, the youngest was selected to participate, and in the case of twins, we randomly selected one of them. All tools and questionnaires were pretested in the area and adjusted before use.

### Data collection of household characteristics and anthropometry

Data were collected during home visits using a semi-structured questionnaire with both open- and close-ended questions. The questionnaire was based on previously validated questionnaires (30), with minor language modifications to make it appropriate for the study participants. It was administered to the lead caregiver through interview and included 1) socio-demographic characteristics; 2) morbidity, if their infants had any illness in the previous 2 weeks or if the infants were sick at the time of data collection; and 3) child feeding practices. Field assistants (with minimum qualification of ‘Advanced level of education’) had 5 days of intensive training in data collection instruments and the collection of anthropometric data using digital scales, tapes, length boards, and measurement techniques as per standardized World Health Organization (WHO) guidelines (31). A different team, composed of nutritionists (bachelors’ degree holders) led by a clinical psychologist (the second author), was trained in administering the child development tools and scoring the performance of the children.

Sanitation was evaluated by observation and recording the presence or absence of stagnant water in the compound, human faeces around the house/veranda, animal droppings, and litter in the compound. If any of these were present, the score would be 0; if none was found, the score would be 1. The presence or absence and the condition of the plate stand (drying rack), bath shelter, and latrine were also obtained by observation. These were scored as good=3, fair=2, poor=1, and none=0. The scores were then combined to make the composite variable (household sanitation) with scores ranging from 0 to 12.

Nutritional status was evaluated using weight, length, head circumference (HC), and mid-upper arm circumference (MUAC), which were measured using standard procedures and calibrations recommended by WHO (31, 32). Anthropometric measurements were performed by a pair of trained field workers. Weight (to the nearest 0.1 kg) was measured with a Seca-scale (model 881, Hamburg, Germany); recumbent length was measured (to the nearest cm) with a length board (SO114530) (33); HC was measured with a non-stretchable tape; MUAC was measured with a non-stretchable tape at the midpoint between the acromion and the olecranon. The child's date of birth was obtained from child health cards. For those who did not have child health cards (8.3%), a record of events was used to determine the approximate date of birth. The anthropometric data including age in months were calculated and converted to *z*-scores (LAZ, weight-for-age *z*-scores (WAZ), weight-for-length *z*-scores (WLZ), mid-upper arm circumference *z*-scores (MUACZ), and head circumference *z*-scores (HCZ) by use of the software Anthro package (version 3.2.2), a nutritional assessment tool using the WHO standards (31, 34). Undernutrition (stunting, underweight, and wasting: LAZ, WAZ, and WLZ, respectively) was defined as a *z*-score less than two SD below the mean on the WHO reference standards (31, 35, 36). A score of less than three SD below the mean on the WHO reference standards of either LAZ, WAZ, or WLZ was defined as severe stunting, underweight, and wasting, respectively.

The socio-economic status of the households was obtained by the ‘Simple Poverty Scorecard for Uganda’ (37), which has 10 indicators that are strongly associated with poverty, and is sensitive to changes in poverty status. The scores were summed up and associated with poverty likelihoods via the ‘National 100% look up table’, a scale that ranges from 0 to 100 (least to most likely to be below the poverty line) (37). The households were then categorized as extreme (47.9–100%), moderate (2.9–47.8%), or least (0–2.8%) likelihood to be below the poverty line.

Infant feeding practices were determined by asking mothers about the first food/drink that was given to the infant, the number of times the infant was fed, the person that normally fed the infant, and the combination of foods normally given. The child dietary diversity scores (CDDS), adapted from the Household Diversity Score tool (38), were used to determine the quality of the individual child's diet (39–43). The tool has a range of 0–8 food groups: 1) grains, roots, or tubers; 2) vitamin A-rich plant foods; 3) other fruits or vegetables; 4) meat, poultry, fish, and seafood; 5) eggs; 6) pulses, legumes, and nuts; 7) milk and milk products; 8) foods cooked in oil or fat. If the child consumed any of the foods the previous 24 h that belonged to any of the group, it would be scored 1, and these were summed up to give the CDDS. The CDDS was then categorized as 0–3 low or ≥ 4 high (39). Previous studies showed that CDDS has a good sensitivity and specificity (40) and correlated significantly with WAZ and LAZ (42).

The Household Food Insecurity Access Scale (HFIAS), without any modifications, was used to determine household food insecurity including all nine generic questions that require a recall about the worry of food availability and accessibility in the previous 4 weeks (44, 45). If the respondent answered ‘yes’ to an occurrence question, a follow-up question was asked to determine whether the condition occurred rarely (once or twice), sometimes (3 to 10 times) or often (more than 10 times). The responses determined the Household Food Insecurity Access Prevalence (HFIAP) status indicator as a proxy of household food insecurity prevalence (44). Each household was then classified into either food secure, or mildly, moderately, and severely food insecure, based on FANTA's recommended cut-offs.

### Cognitive, language, and motor development assessments

Infant development was determined by the BSID III (46) on three sub-scales of cognitive, language, and motor functions. The Bayley scales are known to be the most comprehensive development measures for children up to 42 months (46–48). The BSID III scales were translated to the local language and back translated to English. Furthermore, some pictures in the stimulus and picture booklets were replaced by familiar similar objects in the local setting, for example, apples for tomatoes. The raw scores were converted to composite scores according to the reference material. The mean score is 100 with an SD of 15. Scores below 70 (i.e. <2 SD below the mean for a US reference population) indicate developmental delay.

The infant development was also assessed using the Ages and Stages questionnaires (ASQ) (46, 49–51). The scores obtained from each developmental domain were calculated according to a scale from 0 (worst) to 60 (best), and each child was classified into ‘normal’, ‘delayed’, or ‘needs attention’ based on cut-off values for each of the five domains (48, 52). Both tools were used because ASQ is a caregiver report that captures and establishes a wide range of adaptive behaviours (53) that are not achieved using the BSID III. A 6-day intensive training session was conducted by three research assistants (bachelor's degree holders) and a nutritionist (first author) on the use of the BSID III and ASQ. Data quality assurance was done by the clinical psychologist, who supervised the administration of tests and checked on interview styles and data records throughout the study.

The administration of the tests was done by personnel fluent in English and local language to enhance adequate communication with the children and the caregivers. Administration instructions of time, reversal, and discontinuation rules were strictly followed. Feedback on administration styles and data records were reviewed at the end of each assessment day. Social-emotional and adaptive behaviour subtests of the BSID III were not used due to time constraint and some of these aspects were covered by the ASQ. Inter-rater results showed good correlation (*r*=0.71–0.87) for both scales.

### Statistics

Chi-square and Pearson's correlation tests were used to determine associations of independent variables and nutritional status and the interrelations of other variables, as well as associations between nutritional status with milestone development (aspects of cognitive, language, and motor development). All putative determinants from bivariate analysis were simultaneously included in the various models. In order to identify the most significant determinants, a linear regression analysis was performed using the enter procedures, retaining only the strongest determinants. The variables that did not explain additional variance were not included in the final regression models; all necessary assumptions were met. Variables that showed a significant (*p*<0.05) association in bivariate analyses were included in linear regression models for the different nutritional status indicators to determine the predictors of the dependent variables (Supplementary Table 1). Linear regression was used to examine the relationship between nutritional status and composite scores of cognitive, language, and motor development in children taking into account other factors. The statistical analyses were performed using SPSS version 22 (54) and the level of significance was set at *p*<0.05.

**Table 1:** Household socio-demographic and maternal characteristics (*n*=512)

Characteristics			
HH size (mean [SD])	5.5 (2.1)	Household food insecurity	
3–5	289 (56.4)	Food secure	76 (14.8)
6–13	223 (43.6)	Mild food insecurity	41 (8.0)
HH head age (years) (mean [SD])	32.3 (9.7)	Moderate food insecurity	221 (43.2)
19–29	217 (42.4)	Severe food insecurity	174 (34.0)
30–40	169 (33)	Maternal characteristics	
≥41	99 (19.6)	Maternal age (years) (mean [SD])	26.6 (6.3)
HH head education (years) (mean [SD])	6.2 (3.7)	Maternal education (years) (mean [SD])	4.9 (3.4)
None	58 (12)	None	98 (19.6)
1–6	190 (39.2)	7–10	126 (25.2)
7–10	159 (32.9)	1–6	242 (48.4)
≥11	77 (15.9)	≥11	35 (7.0)
HH head occupation		Maternal occupation	
Peasant farmers	359 (70.1)	Peasant farmers	401 (78.3)
Casual labourers	70 (13.9)	Housewife	86 (16.8)
Business	29 (5.8)	Salary employed	10 (2.0)
Salary employed	27 (5.4)	Others (e.g. business and students)	15 (3.0)
Sanitation in HH		Maternal age at first child (years) (mean [SD])	19.6 (2.8)
Poor sanitation	53 (10.4)	<18	95 (18.9)
Fair sanitation	270 (52.7)	≥18	404 (81.6)
Good sanitation	189 (36.9)	Number of children per mother (mean [SD])	3.4 (2.3)
Likelihood to below poverty line		1–4	371 (72.4)
Extreme likelihood (47.9 to 100.0%)	37 (7.2)	5–8	124 (24.2)
Moderate likelihood (2.9 to 38.1%)	398 (77.9)	9–11	17 (3.4)
Least likelihood (0.0 to 0.8)	77 (15.9)	Mean child age (months) (mean [SD])	7.3 (0.9)

Values are n (%) unless otherwise stated. HH, household.

### Approvals

The study was reviewed by the Makerere University School of Public Health, Higher Degrees Research and Ethics Committee (no. IRB000353) and approved by the Uganda National Council for Science and Technology. It was also approved by the Norwegian Regional Committee for Medical and Health Research Ethics (no. 2013/1833). All respondents gave informed consent by reading and signing a consent form to participate in the study and allowing publication of the findings. The consent form was designed and translated into local language for the participants.

## Results

### Socio-demographic characteristics

All 536 households that were identified consented to participate. Then, 24 households were excluded, mainly due to incorrect reporting of children's ages, leaving a total of 512 households. Table 1 summarizes the household characteristics. The mean (± SD) household size was 5.5±2.1 and most (97%) of the households were reported to be headed by males. The mean age of household heads was 32.3±9.7 years, and they had on average spent 6.2±3.7 years in school. The majority (70%) were peasant farmers. General sanitation in households was fair although a substantial proportion (10.4%) lacked basic sanitary facilities such as latrines, bath shelter, and a plate stand. In some places, these amenities were present, but in poor condition. Most households suffered from moderate to severe food insecurity (43.2 and 34%, respectively). Furthermore, the mean poverty score for all households was 47.7±11.9. Most households (77.9%) had a moderate likelihood of being below the poverty line.

### Maternal and child characteristics

The mean (± SD) number of years a mother had spent in school was 4.9±3.4, and most of the mothers were peasant farmers. Mean age of mothers was 26.6±6.5 years, and the mean age at first birth was 19.6±2.8 years. The mean number of children per mother was 3.4±2.3, and 3.4% of the mothers had more than eight biological children (Table 1). The proportions of boys and girls were about equal (50.8%) and (49.2%), respectively, and their mean age was 7.3±0.9 months.

### Child morbidity

During the last 2 weeks prior to the survey, 65.0% of the children had suffered various illnesses, and 32.4% were still sick at the time of survey. The most common illnesses were cough and flu (56.9%), diarrhoea (26.6%), and fever (sometimes referred to as malaria; 10.3%). Other illnesses reported were skin rash, sore throat, nose bleeding, and eye infections (data not shown). There were no significant differences in gender morbidity (Supplementary Table 1).

### Infant feeding

Most of the index children (97%) were still breastfeeding, and many of them (96%) had started complementary feeding. About 70% of the mothers reported having given only breast milk to their babies up to 6 months, although some of them continued beyond that point, while others had not started complementary feeding at the time of survey (Table 2). Moreover, about two-thirds of the children were reported to be breastfed on demand. There was limited use of infant formula (0.6%) and sources of animal protein.

**Table 2:** Breast feeding and complementary feeding (*n*=512)

Characteristics	*n* (%)
Exclusive breastfeeding first 6 months^a^	357 (70)
Breast feeding frequency	
Whenever the child wants (on demand)	342 (69.0)
Five to eight times per day	143 (29.0)
Two to four times per day	10 (2.0)
Age at introduction of first foods	
Between three and five months	38 (27.0)
At six months	277 (54.0)
After six months	67 (13.1)
Not yet started	19 (3.7)
Child diet diversity score	
Low CDDS (0–3 food groups)	317 (62.0)
High CDDS (4–7 food groups)	194 (38.0)
Combinations of foods commonly fed to children	
A carbohydrate source^b^ and beans	144 (32.6)
Porridge alone or a carbohydrate source alone	138 (31.2)
Beans alone or beans with green vegetable	77 (17.4)
Milk, eggs, silver fish, soy	20 (4.5)
Bean soup	20 (4.5)
Beans, carbohydrate source, green vegetable	15 (3.4)
A source of animal protein^c^ and carbohydrate	14 (3.2)
Green vegetable, fruit alone	6 (1.4)
A source of animal protein^c^, carbohydrate source, green vegetable	5 (1.1)
Porridge with milk	3 (0.7)

a Mothers were asked the age at which they first gave drink or food to the infant.

b This is a group of staple foods including potatoes, sorghum, millet, yams, cassava, maize meal, and rice.

c Foods such as meat, milk, fish, and eggs.

CDDS, child dietary diversity scores.

A majority of the children were first introduced to porridge alone. The combination of foods commonly given to children was beans and a carbohydrate source (e.g. potato, green banana, rice, yam, or maize meal), or a carbohydrate source alone, as reported by approximately one-third of the respondents in each case. The mean (± SD) CDDS was 3.1±1.7 (range 0–8). Few children (4.5%) were fed foods of animal origin (Table 2).

### Growth and cognitive function

The means (SD) of WAZ, LAZ, and WLZ were −0.67 (1.1), −1.14 (1.2), and 0.13 (1.2), respectively. The prevalence of underweight, stunting, and wasting in all children were 12.1, 24.6, and 4.7%, respectively (Table 3). Undernutrition was significantly higher in boys than girls as shown by stunting (30.2% vs. 18.8%), underweight (15.3% vs. 8.8%), and wasting (6.5% vs. 2.8%) (Supplementary Table 1).

**Table 3:** Child nutrition and development status

		*n* (%)	*n* (%)	*n* (%)
Growth indicators	Mean (SD)	<−3.00 SD	−2.99 to −2.00 SD	<−2.00 SD
Weight-for-age *z*-score	−0.68 (1.1)	19 (3.7)	42 (8.2)	61 (11.9)
Length-for-age *z-*score	−1.14 (1.2)	50 (9.8)	76 (14.8)	126 (24.6)
Weight-for-length *z*-score	0.13 (1.2)	8 (1.6)	16 (3.1)	24 (4.7)
MUAC *z*-score	0.28 (1.0)	1(0.2)	10 (2.1)	11 (2.3)
Head circumference *z*-score	0.63 (1.1)	1(0.2)	13 (2.5)	14 (2.7)
Child development scores^a^	Mean (SD)			
BSID II		< −3.00 SD	−2.99 to −2.00 SD	<−2.00 SD
Cognitive scores	102.65 (13.4)	0 (0.0)	6 (1.3)	6 (1.3)
Language composite scores	101.85 (14.7)	1 (0.2)	5 (1.1)	6 (1.3)
Motor composite scores	104.38 (14.7)	4 (1.1)	3 (0.7)	7 (1.8)
Ages and Stages Questionnaire	Mean (SD)	Needs attention	Delayed	
Communication ability	47.31 (12.0)	75 (16.6)	34 (7.5)	
Fine motor ability	53.39 (10.2)	21 (4.7)	20 (4.4)	
Gross motor ability	47.17 (12.8)	82 (18.1)	32 (7.1)	
Problem solving	52.20 (12.2)	31 (7.0)	26 (5.8)	
Personal social ability	50.40 (12.5)	36 (8.0)	32 (1.7)	

A *z*-score of <−3.00 SD indicates severe undernutrition, whereas *z*-scores between −2.99 and −2.00 SD indicate moderate undernutrition.BSID III, Bayley Scales of Infant and Toddler Development third edition.

A score of <−3.00 SD indicates severe development delay, whereas a score between −2.99 and −2.00 SD indicates moderate development delay.

a *n*=456, that is, less than the 512 enrolled children. As a result, some mothers could not be traced for the children to do tests. In some cases, the children would become irritable and testing had to be discontinued.

MUAC, mid-upper arm circumference.

The mean (± SD) composite scores for cognitive, language, and motor development based on the BSID III were 102.6±13.7; 101.8±14.8, and 104.4±15.2, respectively. There were no severely cognitively impaired children, but the language and motor scores indicated that one (0.2%) and four (1.1%) of the children had severe impairment in the two domains, respectively. Results from the ASQ indicated delayed development in all five domains of communication, fine motor, gross motor, problem solving, and personal social ability (Table 3). There were no significant gender differences regarding impairment in cognitive, motor, or language development.

### Association between various characteristics and child nutritional status

The results of the bivariate analysis using the chi-square statistics showed that bigger households and those in extreme likelihood of being below the poverty line were likely to have more underweight children (*p*<0.05; Supplementary Table 1). Household heads with 0–7 years of school were likely to have more stunted children compared to households whose head had more years of school (*p*<0.01); similarly children with low CDDS were more likely to be stunted than those with higher CDDS (*p*<0.05).

Household head education was positively and significantly associated with all the nutritional status indicators, whereas maternal education was significantly associated with only WAZ and WLZ (Supplementary Table 2). There was a negative and significant association between household size and WLZ as well as MUACZ. Child birth order was negatively and significantly associated with WAZ and WLZ. The CDDS were positively and significantly associated with WAZ and LAZ. Poverty likelihood scores were negatively and significantly associated with WAZ, WLZ, HCZ, and MUACZ. Morbidity did not show any significant association with any of the indicators of nutritional status (*p*>0.05).

The linear multivariate regression analyses indicated that the sex of a child was a main predictor of WAZ, followed by sanitation (*R*^2^=0.068) (Table 4). Moreover, the sex of a child and CDDS were predictors of LAZ, while WLZ was predicted by likelihood to be below the poverty line (*R*^2^=0.039 and 0.050, respectively).

**Table 4:** Multivariate linear regression analyses of nutritional status and putative predictors

	WAZ	LAZ	WLZ
*R*^2^	0.068	0.039	0.052
Sex (1=boy)	−0.26* (−0.46 to −0.062)	−0.25* (−0.47 to −0.036)	−0.14 (−0.35 to 0.071)
Household size	–	0.027 (−0.030 to 0.084)	–
Household head education	0.020 (−0.017 to 0.057)	0.018 (−0.023 to 0.058)	–
Maternal education	0.023 (−0.020 to 0.065)	0.016 (−0.029 to 0.062)	–
No. of children	−0.018 (−0.068 to 0.032)	–	−0.047 (−0.099 to 0.005)
Sanitation	0.064* (0.009 to 0.119)	0.039 (−0.021 to 0.099)	0.057 (−0.002 to 0.116)
Poverty likelihood	−0.005 (−0.013 to 0.002)	0.003 (−0.005 to 0.011)	−0.011* (−0.019 to −0.003)
CDDS	0.039 (−0.022 to 0.099)	0.086* (0.020 to 0.151)	−0.036 (−0.100 to 0.028)

Values are B unstandardized regression coefficients (95% CI). Sex (categorical variable) was converted to a dummy variable (0=girl; 1=boy). The rest of the variables were continuous.

*R*^2^, *R*-squared/coefficient of determination; CDDS, individual dietary diversity scores.

* *p*<0.05.

### Nutritional status and child development domains

Supplementary Table 3 shows results of nutritional status indicators’ bivariate correlations with the BSID III and ASQ indicators. Most of the nutritional status indicators were positively (*p*<0.05) associated with language, cognitive, and motor domains (BSID III). However, the correlations between WLZ, HCZ, and cognitive domain were not significant. In the same way, using the ASQ, most of all the nutritional status indicators were positively (*p*<0.05) associated with the development domains, except for LAZ with fine motor and HCZ with personal social abilities, where the associations were not significant.

Multiple regression (using BSID III) showed that the cognitive domain was predicted by WAZ, the sex of a child (boy), and household head education (*R*^2^=0.104) (Table 5). Furthermore, the language domain was predicted by both WAZ and LAZ (*R*^2^=0.068), whereas the motor domain was predicted by the sex of a child (boy), WAZ, LAZ, and likelihood of being below the poverty line (*R*^2^=0.086).

**Table 5:** Nutritional status and independent variables with BSIDIII developmental domains

	Cognitive scores *n*=456	Language scores *n*=456	Motor scores *n*=456
*R*^2^	0.104	0.068	0.086
Sex (1= boy)	3.62* (1.07 to 6.17)	1.69 (−1.02 to 4.40)	3.34* (0.60 to 6.07)
Household head education	0.54* (0.077 to 0.99)	0.18 (−0.27 to 0.63)	–
WAZ scores	3.44** (1.62 to 5.26)	1.75* (0.26 to 3.25)	2.45* (0.48 to 4.41)
LAZ scores	0.29 (−1.00 to 1.59)	1.49* (0.10 to 2.88)	1.46* (0.062 to 2.89)
Sanitation	0.38 (−0.30 to 1.07)	–	–
Maternal age	0.091 (−0.16 to 0.34)	**–**	−0.11 (−0.32 to 0.11)
CDDS	–	0.46 (−0.35–1.27)	–
MUAZ *z*-score	−0.61 (−2.04 to 0.81)	–	−0.72 (−2.25 to 0.82)
Poverty likelihood	0.032 (−0.016 to 0.13)	–	−0.098* (−0.18 to − 0.013)
Maternal education	−0.36 (−0.89 to 0.17)	–	–
Birth order	−0.48 (−1.25 to 0.30)	−0.30 (−0.92 to 0.33)	–

Values are B unstandardized regression coefficients (95% CI).

*n*=456 less than 512 enrolled children. Some mothers could not be traced for the children to do tests and some children would become irritable and tests had to be discontinued.

Sex (categorical variable) was converted to a dummy variable (0 = girl; 1 = boy). The rest of the variables were continuous.

*R*^2^, *R*-squared/coefficient of determination; CDDS, child dietary diversity score.

* *p*<0.05

** *p*<0.01.

## Discussion

### Anthropometry and nutritional status

Nutritional status indicators showed growth faltering as in many other developing countries (55). Boys were particularly more at risk of undernutrition than girls. The trend of a higher level of stunting among boys compared to girls was also observed by UDHS (41% vs. 36%) (1); however, in this study, the margin was higher (30.3% vs. 18.8%). Our findings indicated higher stunting levels (24.6%) compared to the national level (12.4%) for 6- to 8-month-old children (1). However, the levels of underweight and wasting in the current study were lower (11.9 and 4.7%, respectively) than the national level (19.1 and 13.6%, respectively) (1). This finding confirms the previously found high stunting level in southwestern Uganda (41.7%) compared to the national level (33.4%) (1, 11, 14). Evident growth restriction at a young age as 6–8 months old may indicate that growth faltering starts earlier than 6 months (6–8, 10, 11), even as intrauterine growth restriction.

We noted that complementary feeding practices were poor. Some mothers (16.8%) had not started complementary feeding, and the majority (68%) of children had their first complementary food as diluted porridge nothing added. At the time of study, the proportion of children who were being fed foods containing primarily carbohydrate sources and beans was 32.6%; for carbohydrate sources alone, it was 32.6%. The consumption of animal protein foods was limited (4.5%), as also shown in another study (14), and a good combination of sources of animal protein, carbohydrates, and green vegetables was rare (1.1%). There was no mention of the consumption of fat or even the use of fat in cooking. These kinds of diets do not adequately satisfy the nutrient needs (both macro- and micronutrients) of the growing infant, as required by complementary feeding guidelines (12). This was supported by a mean CDDS of 3.1, which is lower than that recommended (11, 14, 39, 56). Indeed, low CDDS was significantly associated with stunting and underweight in bivariate analyses and was also a predictor for stunting (42). Low CDDS could be attributed to high levels of poverty, as shown in one study in southwestern Uganda (14), but also to food insecurity, as shown in this study. It was observed that mothers spent most of the day in the field cultivating and only packed leftover food or porridges for the children who had to come along. The heavy workloads of women and the poverty of Sub-Saharan Africa undermine the nutritional status of mothers, which eventually affects the nutritional status of their children (57).

The significant association between the nutritional status of children and household characteristics related to sanitation, poverty, size, and maternal and household head education children are consistent with UNICEF's conceptual framework of the underlying causes of under nutrition (11, 14). Furthermore, in multivariate linear regression, gender and sanitation were predictors of WAZ. The observed predictors of nutritional status in the various models (sanitation, CDDS, and the likelihood of being below the poverty line) impact nutrition through their interaction with disease, the lack of purchasing power for nutritious foods that cannot be locally obtained, and the lack of knowledge about nutritious foods to give children.

Maternal age at the first child and number of children did not show significant associations with child nutritional status. It is, however, worth noting that some of the mothers were very young (11–16 years) at their first child and that 18.9% were younger than 18 years old and 3.4% had more than eight children. The negative and significant association between household size with WLZ and MUACZ probably indicates inadequate food supply for the larger households and possibly the lack of time to prepare food for infants. Collectively, these factors may have an effect on the health of the mothers, both physically and/or psychologically, that could interfere with child care, including feeding (57, 58).

### Cognitive, language, and motor development versus nutritional status

Cognitive and other development domains correlated positively and significantly with almost all the nutritional status indicators. This is in line with the fact that adequate nutrition is paramount to the development of the brain directly for structural and functional development, and indirectly for children's behaviour and experience (20, 23). Good nutrition is also required for normal physical development of the body. Though both length-for-age and weight-for-age contributed to the model predicting cognitive function, length-for-age did not show significant contribution. Previous studies (15, 16, 59) have shown length-for-age as a strong predictor for cognitive function. We noticed that the boy gender made significant positive contribution in the models predicting cognitive and motor domains.

The findings of this study indicate that the infants’ HCs were adequate and only 2.7% of the infants had HCZ <−2 SD. Studies have shown that HC <−2 SD may be an indicator of severe undernutrition in the first year of life that may eventually affect brain development (60) and hence cognitive function (60–62). Despite the high levels of undernutrition observed in this study, the HCs were within normal ranges, probably because the infants were young (6–8 months). The adequate HCZ could possibly explain the observed good cognitive composite scores (only 1.3% had <−2 SD on the BSID III cognitive scale). However, many of the infants scored below the cut-off values given in the ASQ tool. These scores may be the result of undernutrition as well as lack of time for play, interaction with other children, and stimulation by the mothers. This concurs with the available evidence on the effect of stimulation on child development (17, 18, 63). An early child development intervention of play and communication among caregivers was found to improve mother–child interaction, caregiving environment, and practices that improved feeding and development in children (26, 63).

The two scales (BSID III and ASQ) showed differences in detecting developmental delay evaluated as proportions of low scores. ASQ is a caregiver report that captures a wide range of adaptive behaviours (53) that BSID III does not measure. Still, the two scales showed a low, albeit significant overall agreement (Pearson correlation coefficient=0.37). This finding is similar to a previous study that compared the validity of the two scales and found modest agreement (Pearson correlation coefficient=0.56) (64). Moreover, the observed low values of R^2^ in linear regression models suggest that several variables, though not statistically significant, each had a small impact on our outcomes. It is also possible that other variables, not included in the regression model or study, had significant impact on the outcomes.

### Strengths and limitations

This study has several strengths. It was the first of its kind to use both the BSID III scales and ASQ on children of 6–8 months in the Ugandan setting, and to explore the possibility of correlating these findings to various indicators of nutritional status. We were also able to recruit a large number of children. The main limitation of this study was the cross-sectional design, precluding any conclusion about possible cause and effect. Moreover, neither BSID III nor ASQ has been validated for use in the Ugandan setting. Some of the BSID III tool kit materials were novel to the children and could have intimidated them. Also it was difficult to fit good models that could explain the outcomes in our study population, probably indicating a complex network of variables affecting the outcomes. These findings indicate a need for multi-intervention programmes.

## Conclusion

Undernutrition and growth faltering had already set in as early as 6–8 months among children living in impoverished rural Uganda. Poor household sanitation, poverty, and low dietary diversity, among other factors, were associated with undernutrition in complex interrelationships. Development domains (cognitive, language, and motor) were positively and significantly predicted by the nutritional status of the children confirming previous studies. There is a need for multi-intervention programmes addressing dietary diversity, with food hygiene, infant feeding, and care practices to improve infant and child growth as well as development.

## Supplementary Material

Nutritional and developmental status among 6- to 8-month-old children in southwestern Uganda: a cross-sectional study
