# Supplementary material for: Nutritional and developmental status among 6- to 8-month-old children in southwestern Uganda: a cross-sectional study
Source: Food Nutr Res. 2016 May 27;60:10.3402/fnr.v60.30270. doi: 10.3402/fnr.v60.30270 (PMC4884678; doi:10.3402/fnr.v60.30270)
Supplement: Nutritional and developmental status among 6- to 8-month-old children in southwestern Uganda: a cross-sectional study [file FNR-60-30270-s001.doc]

***Supplementary Table 1.* Bivariate Chi- square associations of independent variables with nutritional status**

**Factors Underweight *p-value* Stunting *p-value* Wasting *p-value* Low MUAC *p-value* Small HC *p-value***

**Child sex**

Male 40 (15.3) 0.03* 79 (30.2) 0.001** 17 (6.5) 0.05 8 (3.1) 0.65 8 (3.1) 0.65

Female 22 (8.8) 47 (18.8) 7 (2.8) 3 (1.2) 6 (2.4)

**Household size**

3-5 people 23 (8.0) 0.001** 68 (23.5) 0.52 11 (3.8) 0.28 5 (1.7) 0.45 8 (2.8) 0.96

6-13 people 39 (17.5) 55 (26.0) 13 (5.8) 6 (2.7) 6 (2.7)

**HHH education**

0-7 yrs in school 48 (13.4) 0.16 100 (28.0) 0.001** 16 (4.5) 0.42 7 (2.0) 0.79 1 (3.1) 0.96

>7 yrs in school 11 (8.7) 19 (15.0) 8 (6.3) 3 (2.4) 3 (2.4)

**CDDS**

Low CDDS 54 (12.9) 0.22 112 (26.9) 0.01* 16 (4.8) 0.77 9 (2.2) 0.97 11 (2.6) 0.78

High CDDS 8 (8.4) 14 (14.7) 5 (5.3) 2 (2.1) 3 (3.2)

**Morbidity at the time of survey**

Sick 23 (13.9) 0.32 40 (24.1) 0.90 9 (5.4) 0.54 3 (1.8) 1.00 4 (2.4) 0.72

Not sick 18 (10.3) 43 (24.7) 7 (4.0) 3 (1.7) 3 (1.7)

**Breast feeding frequency**

On demand 44 (12.9) 0.32 86 (25.1) 0.37 15 (4.4) 0.69 6 (1.8) 0.53 5 (1.5) 0.02*

< 8 times/ day 15 (9.1) 33 (21.4) 8 (5.2) 4 (2.6) 8 (5.2)

**Exclusive breast feeding**

Yes 20 (13.4) 0.56 33 (22.1) 0.41 7 (4.7) 0.99 3 (2.0) 0.89 5 (3.4) 0.58

No 42 (11.6) 93 (25.6) 17 (4.7) 8 (2.2) 9 (2.5)

**Poverty likelihood**

Extreme 9 (24.3) 0.01* 10 (27.0) 0.23 4 (10.8) 0.08 3 (8.1) 0.02* 2 (5.4) 0.59

Moderate 49 (12.3) 103 (25.9) 19 (4.8) 8 (2.0) 10 (2.5)

Least 4 (5.2) 13 (16.9) 1 (1.3) 0 (0.0) 2 (2.6)

**Household sanitation**

Poor 27 (24.8) 0.001** 35 (32.1) 0.09 8 (7.3) 0.13 5 (4.6) 0.07 5 (4.6) 0.21

Fair 19 (8.3) 55 (24.0) 12 (5.2) 5 (2.2) 7 (3.1)

Good 16 (9.2) 36 (20.7) 4(2.3) 1 (0.6) 2 (1.1)

Values are numbers (percentages); **p* < 0.05; ** *p* < 0.01; HHH – household head; CDDS – child diet diversity score.

***Supplementary Table 2.* Bivariate correlations of independent variables with nutritional status of the children**

Associated factor WAZ LAZ WLZ MUACZ HCZ

^a^Child sex -0.12** -0.11* -0.06 -0.07 -0.14**

HHH education 0.16** 0.11** 0.10* 0.12** 0.11*

Maternal education 0.12**0.06 0.10* 0.10 0.10

Household size -0.10 0.05 -0.14** -0.18** -0.05

Birth order -0.11* 0.02 -0.12** 0.15 -0.03

CDDS 0.12** 0.15** -0.01 0.08 0.04

Household sanitation 0.17** 0.09* 0.13** 0.10* 0.04

Maternal age 0.12**0.06 0.10* 0.10 0.10

Poverty likelihood -0.16** -0.02 -0.19** -0.15** -0.11*

Exclusive breastfeeding 0.03 0.04 0.00 0.06 0.00

Values are Pearson’s correlation coefficients; **p* < 0.05; ***p* < 0.01; ^a^Sex (girl= 0 and boy = 1).

WAZ – weight-for-age z-score

LAZ – length-for- age z-score

WLZ – weight-for-length z-score

MUACZ - mid-upper arm circumference z-score

HCZ - head circumference z-score

HHH - household head

CDDS – child dietary diversity scores

***Supplementary Table 3.* Associations between nutritional status and child development**

| **BSID III (bivariate correlation) Ages and stages questionnaires (bivariate correlation)**  **--------------------------------------------- ----------------------------------------------------------------------------------**  **Language Cognitive Motor Commu- Gross motor Fine motor Problem- Personal-**  **nication solving social**  **Nutritional**  **status ^a^***n*=456 *n*=456 *n*=456 *n*= 453 *n*=452 *n*=450 *n*=446 *n*=448  **Pearson’s correlation coefficient values** |
| --- |

WAZ 0.27** 0.22** 0.24** 0.14** 0.30** 0.18** 0.23** 0.20**

LAZ 0.18** 0.19** 0.20** 0.04* 0.17** 0.05 0.14** 0.13**

WLZ 0.16** 0.08 0.11* 0.12* 0.19** 0.17* 0.14** 0.13**

MUACZ 0.17** 0.13** 0.12** 0.11* 0.23** 0.12** 0.21** 0.14**

HCZ 0.15** 0.08 0.12** 0.10* 0.22** 0.14** 0.20** 0.09

|  |
| --- |

**p* < 0.05; ***p* < 0.01

WAZ – weight-for-age z-scores

LAZ – length-for-age z-scores

WLZ – weight-for-length z-scores

MUACZ – mid upper arm circumference z-scores

HCZ - head circumference z-scores

*^a^n* = 446-456, i.e. less than the 512 enrolled children. Some mothers could not be traced for the children to do tests and some children would become irritable and testing had to be discontinued.
